# “I have never experienced any problem with my health. So far, it hasn’t been harmful”: older Greek-Australian smokers’ views on smoking: a qualitative study

**DOI:** 10.1186/s12889-015-1677-6

**Published:** 2015-03-29

**Authors:** Masoud Mohammadnezhad, George Tsourtos, Carlene Wilson, Julie Ratcliffe, Paul Ward

**Affiliations:** Discipline of Public Health, Flinders University, GPO Box 2100, Adelaide, South Australia 5001 Australia; Department of Health Education and Promotion, School of Public Health, Tehran University of Medical Sciences, Tehran, Iran; Flinders Centre for Innovation in Cancer, Flinders University, GPO Box 2100, Adelaide, 5001, South Australia Australia; Cancer Council South Australia, Eastwood, South Australia Australia; Flinders Health Economics Group, School of Medicine, Flinders University, GPO Box 2100, Adelaide, 5001, South Australia Australia

**Keywords:** Greek-Australian smokers, Older people, Qualitative study, Knowledge, Attitude

## Abstract

**Background:**

Smoking tobacco products is one of the largest preventable health risk factors for older people. Greek-Australians have the highest prevalence of cigarette use in Australia for older people, but there is a lack of knowledge about Greek-Australian’s perspectives on smoking cessation. The purpose of this exploratory, qualitative study was to progress the knowledge base in this area.

**Methods:**

A qualitative study was designed to gather information on participants’ perspectives about, and understanding of, their reasons for smoking and their attitudes to quitting. A snowball sampling technique was used to identify twenty Greek–Australian current smokers, aged ≥50 years. Semi-structured, face-to-face interviews were conducted with the assistance of a Greek translator. The audio-taped interviews were transcribed and then qualitative content analysis was used to categorise responses to the questions.

**Results:**

Participants’ perspectives on three broad topics were identified in the interviews: perceived benefits of smoking, perceptions of smoking and its effect on health, and barriers to cessation. Smoking behaviour was described as contributing to tiredness, and stress, and yet also was also a source of enjoyment. Level of knowledge about smoking-related diseases and the risks of smoking was very low. The number of cigarettes smoked each day, type of smoking (i.e. pipe rather than cigarettes), and previous family history of smoking were identified as indicators that limited harm flows from smoking. Most participants had a positive attitude towards smoking and described their own life experience and cultural norms as supporting smoking acceptability. Low confidence in quitting was linked to advanced age.

**Conclusion:**

Smoking among older Greek-Australian smokers has been associated with a number of influences and these need to be addressed in smoking cessation efforts targeted at this group. Promoting knowledge about the health impacts of smoking, changing attitudes towards smoking, and ultimately, decreasing tobacco consumption are critical to the maintenance of health among older Greek Australians. Cultural and experiential influences may increase the difficulty associated with changing these outcomes, but may also serve as a framework from which to develop and implement an educational intervention tailored for older Greek-Australians.

## Background

Tobacco smoking is a major preventable cause of disease and premature death for older people [1]. In Australia, in 2010, the prevalence of smoking was reported to be 21.4 percent among people aged 50 to 59 years, 15.4 percent among people aged 60 to 69, and 7.8 percent of those older than 70 years were smokers [2]. The harmful effects of smoking are particularly serious for older people [3], and the mortality rate among older people is double that of non-smokers of similar ages [4]. In older people, smoking is a major contributory factor to a range of diseases including heart disease, stroke, respiratory disease and cancer [5]. Among older smokers, the benefits of quitting in terms of heart disease and stroke are almost immediate, and there is a rapid decrease in rates of mortality [6].

Smoking consumption in older smokers has a number of characteristics that need to be taken into account when designing an intervention to reduce or eliminate smoking in this group. These characteristics include; smoking history, extent of dependence on nicotine [7], the number of previous unsuccessful attempts to quit [8], and smoker doubts about the benefits of cessation. Older smokers may not accept that they are at risk of disease [9,10].

In Australia, different levels of smoking consumption have been reported among different ethnic groups. One study found that 42% of Indigenous Australians aged between 15 to 24 years smoke [11], compared to only 29.7% of Australians with Arabic backgrounds [12].

Greek-born Australians represent one of the largest ethnic communities in Australia. In the 2001 it was estimated that 2.8 per cent of the overseas-born population were Greek and this group constituted 0.6 percent of the total Australian population [13].

Smoking rates for some non-English speaking Background (NESB) groups, including Greek-Australians remains high, even though the prevalence of smoking in Australia in general has declined over the past two decades. Previous studies have highlighted a higher prevalence of smoking among Greek-Australians compared to other ethnic groups. In 1998, a household survey in the Marrickville Local Government Area (LGA) revealed smoking rates among males Greeks were significantly higher than for the general population (43% and 23% respectively). Other studies have shown similar findings in smoking prevalence among Greek-born males [14]. The smoking rate among elderly Greek-Australians is also higher than that of the average elderly Australian; it is estimated at 18.4% for Greek-born Australians aged 70 or older in comparison with 12% among the general Australian population in the same age group of [15].

Health-related knowledge can be an important factor that contributes to disparate views on the causes of smoking-related disease, such as cancer [16]. The results of previous studies among older smokers show that they have different levels of knowledge of, and varying perceptions about, the harms of smoking and the benefits of smoking cessation. For instance, Kerr et al., (2011) utilising interviews with 20 Scottish smokers and ex-smokers aged 60 and over, found that the majority of current smokers were aware of the harmful health effects of smoking, although some of them were not fully aware of the most serious health dangers. Some of them had tried quitting and they indicated that health-related factors provided the motivation to quit [17].

Other influences on individual decision-making about quitting include community norms about the behaviour. Medbø et al., (2011) reported that the attitude of elderly smokers to quitting and not starting again, was highly dependent upon the attitudes of “significant” others towards smoking. In order of importance, these “influencers” were spouse, family and friends [18].

Fifteen years ago it was reported that Greek-Australian smokers not only smoke more than other NESB groups; they have less smoking-related knowledge and less intention to quit smoking compared with other minority groups [19]. Whether this attitude and lack of knowledge persists among older Greek-Australians is unknown. As far as we know, there are very few studies of smoking in the Greek-Australian community in general and there is no study on Greek-Australian older people specifically. Considering the fact that Greeks form one of the main ethnic groups in Australia, and that smoking rates in older Greek-Australians are higher than the average rates for other older Australians, understanding the smoking-related knowledge and perceptions of older Greek-Australians could illuminate effective means to reduce smoking for this group. Eliciting the views of older Greek-Australians about smoking will help provide an understanding of the current influences on smoking behaviour among this ethnic group. Moreover, this information can be used as the basis for designing and developing effective, ethnically-specific smoking preventative strategies which could be useful for both Greeks and other minority groups. Hence, the aim of this study is to gain a greater depth of understanding about older Greek-Australian smokers in relation to their smoking behaviour and their views on smoking cessation.

## Methods

A qualitative study was undertaken with a group of male and female older Greek-Australians in order to gain insights into smoking-related knowledge and underlying attitudes to smoking. Participants were interviewed individually using a face-to-face, semi-structured interview schedule and, where a previous quit attempt had been made, participants were encouraged to ‘tell their stories about smoking cessation’.

### Sample and setting

Participants were current smokers aged 50 years or over who self-identified as Greek-Australians and who resided in metropolitan Adelaide. Smokers were defined as those who had smoked at least 100 cigarettes during his/her lifetime and were currently smoking [20]. They were recruited through the Greek Orthodox Community Centre of South Australia (GOCSA). Participants included both attendees and staff of the centre. To develop aspects of a conceptual category and to reach data saturation [21], 20 current smokers (twelve males and eight females), were recruited in two stages, each of two-month duration over a one-year period. An in-principle letter of support was obtained from the GOCSA manager and the interviews were undertaken in the centre. In the first stage the first ten smokers were interviewed and then their interview transcripts were reviewed. Following analysis and interpretation of the data and extraction of themes, additional interviews were scheduled.

### The interview

A face-to-face interview of 45 to 60 minutes duration was conducted for all the participants by the first author who had experience in conducting semi-structured interviews with the assistance of a nationally accredited translator. The translator translated questions and prompts from English to Greek and responses from Greek to English. Each interview started with an invitation to participants to speak freely about their experience of, and attitude towards, smoking. To understand how data saturation might apply to this study, the researcher reviewed the content of transcribed interviews and also all the codes, concepts, categories, relationships and themes. Near the end of the second round of interviews it became clear that data redundancy was evident and that the categories, themes and inter-relationships had been thoroughly described. All interviews were audio-recorded for the purposes of transcription. Thirty dollars reimbursement was offered to compensate the respondents for time and out-of-pocket expenses associated with their participation.

### Interview schedule and content

An interview schedule was developed based on the relevant literature and the study’s research aims and questions. Semi-structured interviews were used because they have the required flexibility to allow participants to draw on their life experiences when describing their smoking status [22]. The interview schedule was further developed through an iterative process (where interviews and analysis occur in parallel). The iterative process continued until data saturation was achieved [23]. This approach enabled exploration of multiple aspects of the participants’ smoking status. Some questions encouraged participants to reflect on their experiences, a technique known to increase the validity of participants’ responses [24].

The interviews included open-ended questions aimed at exploring the participants’ knowledge, attitudes and beliefs about smoking or matters associated with smoking, including their experience of barriers to quitting. Demographic information including age, marital status, education, employment status and, preferred language were collected. Participants were asked to describe their smoking history, cultural norms, environmental cues and their personal attitudes towards smoking. They were also asked about their understanding of the health-related risks of smoking and the benefits of quitting (see the ‘List of Saints’ section).

- **Summary of the topics discussed during the interview**
- Smoking antecedents (i.e. kind of smoking, starting age of smoking, reasons started, number of years as a smoker, country of starting smoking)
- Relationship between disease occurrence and smoking
- What was good about smoking
- What was bad about smoking — knowledge about benefits of smoking cessation
- Self-confidence and stage of motivation to quit smoking
- Previous quit attempts (i.e. number of attempts, duration of quitting)
- Reasons for relapse in later life and their experience of what led to successful quitting
- Barriers to smoking cessation in later life
- Counselling by health professionals (doctor, psychologist and so on)

### Recruitment and data collection

We recruited older Greek-Australian smokers into the study using a snowball sampling, where individuals identify potential participants known to them. This kind of sampling is useful in qualitative research because the target population required was small, hard to reach and unique according their smoking status and age [24]. We asked early participants to name smokers who may also be interested in participating in the study. After visiting GOCSA and obtaining primary information from the community managers about their previous experience of better engagements and communications with older people, accessing to potential samples, and facilities which GOCSA can provide for the researcher to conduct interviews, it was recognised that most Greek-Australians, even though living in the Australian community, prefer to speak Greek. A bilingual, qualified translator was present during the interviews and all written materials were offered in both Greek and English. These included the consent form, letter of introduction, and information sheet. To ensure the accuracy of translation, all the translated materials were reviewed by another Greek translator. The translator had a great deal of experience of working with Greek-Australian residents, including as a translator for other research. Translation was an active procedure, with the translator playing an intermediary role during the interviews.

### Data transcription and analysis

Transcription of the interview content was conducted in an empty room to eliminate background noise, which might reduce clarity and introduce guesswork into the transcription. To achieve accuracy in the transcription, copies of the transcriptions were compared to samples of the audio recordings of the interviews.

Data analysis was conducted by the main researcher who had previous experience and with the help of research colleagues. Data analysis started after completing the first round (n = 10) of data collection. The transcribed texts were analysed using qualitative content analysis to identify the themes. Content analysis helps the researcher to ‘sanitize’ words into a number of content-related categories and then count the number of instances they fall into each category [25]. The interview data were synthesized under each of the question headings (e.g. ‘What was good about smoking? What was bad about smoking?’).

All text was read and re-read closely by the main researcher to generate codes. The difference in coding was discussed with other research colleagues and some of the codes were adjusted. Key words or phrases that appeared relevant to the research questions were categorized according to content, a process which generated many units of meaning or codes [26]. Codes were then analyzed in terms of their frequency so that their occurrence in each category could be specified and compared (e.g., ‘five of seven interviewees have said…’, ‘the majority of the answers focused…’) [27]. The text that corresponded to how the participant perceived their smoking experiences in different field of factors as categorized in the ‘List of Saints’ section, extracted from the transcripts and categorized a similar answers to each question.

Two stages were involved in the identification of the final themes. At the first stage, less relevant passages and paraphrases with the same meaning were skipped. To achieve greater similarity between the categorized meanings, similar paraphrases were bundled and summarized at the second stage [23].

The researcher used Statistical Package for the Social Sciences (SPSS) version 20 to generate descriptive statistics to illustrate smokers’ education, age, and health status.

### Ethical considerations

This study was approved by the Social and Behavioural Research Ethic Committee (SBREC) of Flinders University, South Australia. Informed consent was gained by providing the participants with a consent form, an introduction letter, and an information sheet in both English and Greek versions. The aim of the study was clearly explained to the participants and they were aware that their participation in the study was voluntary and they could withdraw from the study at any time. All participants were informed that their transcribed information would remain confidential. Transcripts and audio tape recordings were labelled with a code only and no identifiable names were used.

## Results

### Participants’ characteristics

Twenty Greek smokers who were more than 50 years old were interviewed for this study (twelve males and eight females). Their mean age was 64.6 years (SD = 9. 96 years). Most of the participants had completed high school level of education (n = 12) and most of them preferred to communicate in Greek (n = 12) and the same number identified as pensioners. Most (n = 13) were suffering from diseases such as cancer or heart disease (Table 1).

**Table 1:** **General characteristics of participants**

**Participants code/gender**	**Age**	**Education level**	**Preferred language**	**Occupation**	**Health situation**
1. Male	79	Primary school	Greek	Pensioner	3 heart attacks and bladder cancer
2. Male	71	Primary school	Greek	Fitter/welder/blacksmith (Pensioner)	No disease
3. Female	51	High school	English	Work in nursing home (GOCSA)	No disease
4. Male	76	Primary school	Greek	Farmer/laying cement foundations (Pensioner)	Heart surgery twice (coronary obstruction)
5. Male	73	Primary school	Greek	Pensioner	Colon cancer
6. Male	73	Primary school	Greek	Pensioner	No disease
7. Male	74	Primary school	Greek	Gas company (Pensioner)	No disease
8. Male	74	Primary school	Greek	Assembler (pensioner)	Respiratory problems
9. Female	61	High school	Both English and Greek	Translator	Respiratory problems
10. Female	65	High school	Both English and Greek	Pensioner	High blood pressure and hyperthyroid
11. Male	66	High school	Greek and English	Taxi driver	Diabetes and sarcoidosis
12. Male	62	High school	Greek	Pensioner	Back pain
13. Male	51	High school	Greek	Taxi driver	No disease
14. Female	53	Bachelor degree	English	School teacher	High blood pressure
15. Female	70	High school	Greek	Pensioner	Osteoporosis
16. Male	72	High school	Greek	Pensioner	Emphysema and liver problem
17. Male	53	High school	English	Taxi driver	No disease
18. Female	69	High school	Greek	Pensioner	No disease
19. Female	50	High school	English	Bank teller	Hyperthyroid
20. Female	50	High school	English	Unemployed	Crohn’s disease

Nearly all of the participants (n = 17) reported that they smoked cigarettes only and all of them smoked daily. All said that they started smoking during their adolescence or early adulthood. The mean age of smoking commencement was 19 years (SD = 3. 72 years) and 14 of the respondents said that they started smoking when they still lived in Greece. The mean years of smoking were 45.5 years (SD = 10.8 years), and about half of the respondents (n = 11) said that they had smoked for more than 50 years.

The mean number of cigarettes smoked each day was 16.5 (SD = 9.98 cigarettes). Fourteen of the participants indicated that they started smoking within 30 minutes of waking up. Eleven of the participants reported that they had attempted quitting at least twice, with 15 attempts being the maximum. Among the 16 smokers who had tried to quit smoking, at least once 11 had quit for at least three months, (maximum 20 years) (Table 2).

**Table 2:** **Smoking-related characteristics of participants**

**P.C**	**Kind of smoking**	**Starting age/country**	**Years as a smoker**	**Average of cigarettes daily**	**Approximate starting time after waking up**	**Number of quitting attempts**	**Longest period of stopping**
1	Cigarette	22/Greece	57	18-20	5 min	3	20 years
1	Tobacco	17/ Greece	54	20-25	30 min	0	-
3	Cigarette	27/Australia	24	12	30 min	4	2 weeks
4	Cigarette	16/Greece	58	25	2-3 hours	1	2 years
5	Tobacco	12/Greece	61	2-3	30 min	1	3 days
6	Cigarette	22/Greece	51	10-12	20 min	1	b2 weeks
7	aCigarette	19/Greece	55	25-30	20-30 min	0	-
8	Cigarette	24/Greece	50	20	30 min	1	c4 days
9	Cigarette	16/Greece	45	6-10	10 min	10	1 year
10	Cigarette	20/Greece	47	4	10 min	10-15	6 months
11	Cigarette	15/Greece	49	30	5 min	4-5	9 months
12	Cigarette	14/Greece	48	18-20	20 min	2-3	6 days
13	Cigarette	19/Australia	32	25	1-2 hours	0	-
14	Cigarette	18/Australia	30	10	1 hours	3-4	11 years
15	Cigarette	25/Greece	45	2-3	No	2	d3 years
16	Cigarette	17/Greece	55	25	15 min	2	2 years
17	Tobacco	18/Australia	35	30	5 min	3	5 years
18	Cigarette	19/Greece	50	10	30 min	4	d9 Months
19	Cigarette	20/Australia	30	2-3	No	1	3 months
20	Cigarette	20/Australia	35	3-4	No	0	-

Three topics were examined on the basis of material extracted from the participants’ interviews: perceived benefits of smoking, knowledge of smoking and its effect on health, and barriers to cessation.

### Perceived benefits of smoking

When the participants were asked about their reasons for smoking, the majority of the respondents indicated that smoking was “beneficial”; it assisted them to manage tiredness and stress, it helped them to relax, offered enjoyment, and was integrated into their social activities.

P17 (a 53-year-old male) was a taxi driver. He genuinely believed that smoking could reduce his stress:

*I think stress is a big factor. When you have a problem or you are worried you smoke more. It reduces my stress and when I go to the doctor or somebody else and my anxiety builds up I smoke cigarettes. The reasons that I started smoking were that we had a lot of stress. Now, whenever I am under stress I probably light up.*

P2 (a 71-year-old male), started smoking when he was 17 and explained that smoking was part of his social life:

*It is also a social thing. I do like to smoke. When you go out you like to have a coffee and then have a cigarette. It is part of your routine. You have a coffee, you have a cigarette and you have a conversation. You know it is a social part of your life more than anything else, even when at home.*

Three participants indicated that they smoked for enjoyment. P14 (a 53-year-old female), had a mother who smoked all her life. Her father was a smoker and he died because of cancer. P14 explains here that she smokes for enjoyment even though she knows it can damage her health:

*I have no idea. I just enjoy it. I know that smoking is very harmful, but I as I said before, I still enjoy it. Really the best part is the light up for the first puff. Then, you know, when I go home, I rest and I have a cigarette and I look at my garden and smoke a cigarette. I enjoy a cigarette.*

### Knowledge of smoking and its effects on health

The majority of participants (n = 14) were not fully aware of the dangers of smoking, despite acknowledging being exposed to public health messages. Only six smokers were well informed about the dangers of smoking. It is notable that these six participants had a higher level of education in than those with poorer knowledge of the health consequences of smoking. P2 (a 71-year-old male) contended smoking was actually good for his health:

*So far I have never experienced any problem with my health. So far, It hasn’t been harmful […] I can’t stay in places when I can’t smoke. I have been sick whenever I have quit. [When I smoke] I can think better. It improves my breathing and my mental state.*

Of the 13 respondents who suffered from various diseases, eight had smoking-related diseases. When the participants were asked about the relationship between smoking and their disease, four of them denied the potential for this relationship. The other four were uncertain.

^b^(74-year-old male) suffered a respiratory problem but refused to accept any possible relationship between smoking and his symptoms and seemed unaware of the health dangers of smoking:

*I don’t know [the cause of] this trouble. When my phlegm went black I went to the doctor and he checked me over and said there was nothing wrong. It came clear but after four years it happened again. The doctor sent me for an X-ray and it showed that nothing was wrong.*

Eight participants who had potentially smoking-related disease made judgments about the relationship between smoking and their health condition based on their own or their family’s personal experiences. P14 (a 53-year-old female) contended her high blood pressure was hereditary and had nothing to do with her smoking. She further argued that if she was ever diagnosed with cancer it would arise from her familial cancer risk:

*No [I discount any relationship] because I was diagnosed with [high blood pressure] in 2000 and I was not smoking at that time. It is more a hereditary thing and clearly smoking doesn’t affect it. I have smoked a lot of cigarettes in the past because my father was dying of lung cancer and his younger brother also got lung cancer. I probably am a good candidate [for lung cancer] and I could get it because I used to smoke at one stage a lot but now I don’t smoke a lot and so it is not a problem.*

Five of the participants, however, indicated that they realized there were some adverse health side-effects associated with smoking but that the low number of cigarettes they consumed (3 to 12 cigarettes a day), or the kind of tobacco they smoked, meant they were not at risk. P6 (a 73-year-old male) had smoked for about 51 years, ten to twelve cigarettes per day. He believed smoking a “few” cigarettes a day was not a health risk (no risk) and therefore he didn’t need any smoking cessation advice or services:

*I believe that 10 to 12 cigarettes a day is not harmful to me and that is why I haven’t asked for help [to quit]. The amount of cigarettes that I smoke, no, but if you smoke more, yes.*

P5 (a 73-year-old male), started smoking when he was 12. He contended that the kind of tobacco he smoked was not harmful and was free of side-effects:

*Up to now I have never coughed because I have never changed tobacco. I have smoked all the time since 1960. I started off with Drum tobacco and I have never changed. With a Drum smoke no cough, no nothing.*

Three of the respondents said that while they believed that smoking was harmful, they also thought that quitting smoking had no health benefits. To justify this claim, they referred to people who had died of cancer even when they had never been smokers or they said they knew a lot of doctors who smoked cigarettes.

P2 (a 71-year-old male) maintained smoking was not harmful because he knew many non-smokers who had died of cancer.

*Government makes profit from cigarettes and put this advertise on cigarette packets that it makes your health damage, it causes cancer. I don’t believe it. There are a lot of people who have never smoked and who get cancer.*

Eight of the participants acknowledged that quitting smoking would be advantageous to their health, although they did not identify in what way and their decisions seemed mostly influenced by their own previous experiences of smoking-related diseases. For example, they claimed that their decision to quit smoking was caused by their phlegm, breathing problems, cough, headache, or to reduce their eye soreness.

P19’s (a 50-year-old female) knowledge of the benefits of quitting was confined to minor symptoms such as headaches and sore eyes, as she explained below:

*Probably. How do I know? Because I find if I smoke too much I feel tired and my breathing is affected. Well, I feel better when I don’t smoke. If I smoke too much I get headaches and sore eyes. That is how it affects me. Other people don’t get this. Everybody is different.*

Four of the participants stated that smoking cessation was a good idea for younger adults but not for older people. They believed that, among older smokers the damage had already been done and quitting would not be beneficial. For example, P11 (a 66-year-old male) who smoked 30 cigarettes a day indicated some awareness of the health benefits of cessation, but concluded that in his case the damage had been done:

*I think it probably would be beneficial but it is a bit late. I mean I have smoked so long the damage has been done. Even so, it could benefit rather than be harmful.*

Eight of the interviewees believed strongly that quitting smoking did not have any positive effect on health; rather, it could be harmful to one’s health. ^a^ (a 74-year-old male), discounted the benefits of smoking cessation and also had a low perception of risk. He judged that, at his age, with his smoking history, there might actually be more risk in quitting:

*Listen. If you’ve smoked for 55 years like I have you have to run with it. If you quit smoking now, your body may not cope with the change; it might even make you sick in some way.*

Only five of the respondents had a negative attitude towards smoking and believed that smoking was a “crazy habit”. P9 (a 61-year-old female) described her attitude towards smoking withn the following:

*I think we are stupid for smoking. Because we are damaging our health and I am trying very hard to stop, but I don’t succeed.*

Some had a positive attitude towards smoking, particularly notion no identifiable compromises to their health. *‘Smoking doesn’t have any harmful effect on my body (my chest X-ray was clear) so therefore I don’t need to quit smoking’* (P1); and P2 reported: *‘I know a lot of people who didn’t smoke, but they are in the cemetery’*. One of the participants recalled: *’When I went to my doctor’s office, he told me not to smoke but then I saw he was smoking’* (P5). Another rationalized her smoking by pointing out that in her family the smokers are healthy while some non-smokers have cancer:

*In my family all family members who smoke cigarettes are healthy while two of [my wife’s] family members who haven’t ever smoked have cancer.* (P10).

### Barriers to cessation

As shown in Table 2, sixteen of the respondents had stopped smoking at some point in their past, with the duration of cessation varying between less than two weeks and over three months. None of the participants had previous plan to stay quit forever. As a consequence, the majority of the respondents (n = 16) had very low confidence in their capacity to quit smoking with most indicating less than ten percent when asked to specify a percentage. P18 (a 69-year-old female), described an accident that had caused pain in her back and neck and some anxiety and this impacted her confidence. Although she had strong intentions to quit her low self-confidence prevented he succeeding:

*Look, every night I tell myself that I am not going to smoke tomorrow and I must be strong, but next morning when I have my coffee I start smoking again. I am no confidence. My problem is that I have given it up before even for nine months, but I found that it is very struggle every day.*

Of the 16 participants who had attempted to quit, three of them reported that their wife or child’s death was the main reason for their smoking relapse. Others mentioned the following influences on relapse: mental and nervous problems (six), visiting other smokers including friends and family members (six), accessibility to cigarettes (eight), loneliness (two), stress (two), fun (one) and lack of knowledge (one).

P3 (a 51-year-old female), had tried to quit four times with two weeks her longest cessation. She described the powerful effect of withdrawal symptoms and nicotine craving and how these created a barrier to successful cessation:

*I was agitated. I was restless. I felt full of tension. You know my body was just reacting and I found that if I had a coffee I associated it with having a cigarette. If I had a coffee without a cigarette I tended to crave food. It was a case of what do you do with your hands… it’s a similar feeling to being pregnant. That’s why I found it hard to stop.*

The majority of participants (n = 14) agreed that smoking is a habit and an addiction. P2 explains:

*I am addicted to smoking, my body needs nicotine, and this is the main cause of my smoking...... I can turn on to safe activity instead of cigarettes, but that is just your body reaction and you need nicotine and that nicotine, of course is a drug which my body is addicted to it. That is a hard part.*

## Discussion

The findings from this study illustrate that, in general, participants had a low level of knowledge about the harmfulness of smoking and the benefits of smoking cessation. This seems surprising given the prevalence of TV adverts, cigarette packets, billboards and media attention given over to the harms of smoking. They also exhibited a positive attitude towards smoking. These influences on their smoking were mostly related to their personal experiences, life events, habits, and their cultural beliefs. Participants also reported barriers to smoking cessation that included low self-confidence, family challenges and critical life events along with stress and loneliness.

Smoking-related knowledge and attitudes towards smoking are the main predictors of smoking behaviour [28]. Poor knowledge of the health effects may impact on the motivation to change [29]. Similarly, overall attitude towards smoking plays an important role in the initiation and maintenance of smoking [30]. In populations where smokers lack knowledge of the benefits of smoking cessation, and have a positive perception of the benefits of smoking, cessation challenges will be maximized. In the present study, although some of the participants were aware of the general harms of smoking, they were not well-informed about the detail or the extent of the harm caused. This results is consistent with observation from a study of oral health patients in Switzerland: Bornstein et al., (2012) found that current smokers were significantly less aware of the effect of smoking on oral health than others [31]. Among those who were aware of the harmfulness of smoking on general health, most were not aware about the negative effects of smoking on oral health [32].

The results reported here also highlight the importance of addressing, within this population, the lack of perceived benefits from quitting and the perception that smoking-related disease may not be severe of in this population. The situation is further complicated by misapprehension about the health benefits accrued from smoking cessation late in life. Lyna et al., (2002) confirmed that older smokers thought themselves at elevated risk for lung cancer, regardless of whether they continued or quit smoking [33]. This study, and the results presented in this paper, are consistent with the idea of people being ‘candidates’ for future disease based on their past and current health behaviours. Respondents in our study did not perceive themselves as having a high ‘candidacy’ for future illness on the basis of their smoking, thereby reducing their perceived need for smoking cessation [34]. Previous studies have shown that culture relates closely to perceptions of risk so providing health services within a cultural context may increase their effectiveness [35].

Greek smokers in Australia, as an ethnic group, also exhibit some behaviours which are affected by their culture in terms of smoking consumption and quit attempts [36]. Because Greek-Australians have a high collective culture, the participants’ positive attitude towards smoking could be interpreted in the light of their culture, which has traditionally accepted smoking and has few cultural prejudices against smoking [37].

Health behaviour models describe the role of psychosocial risks and how protective factors, like beliefs about the risks and perceived benefits of smoking, predict smoking behaviour [38]. Smokers who perceive the risks rather than the benefits of smoking are more motivated to quit smoking [39]. McKee et al. (2005) found that smokers who perceived the benefits of quitting smoking formed stronger intentions to quit smoking [40].

The results of our study show that the majority of older Greek-Australian smokers in our sample justify their smoking by describing how it helps them to reduce their life stress and to feel relaxed. Addiction to nicotine and being habituated to smoking were also cited as reasons for smoking. Some participants described smoking as a way to increase their enjoyment of life. Many smokers view tobacco use solely as a means to cope with stress and anxiety [41]. There is a known established relationship between daily negative events and stress and smoking [42] with smoking being a ‘rational’ response to daily stress [43].

Smoking also represents a social activity and it appears to act not only as a means of coping with stress and exclusion, but also as a means of expressing identification and belonging.

The results of the present study show that older Greek-Australian smokers have a high sense of perceived barriers due to low self-efficacy or self-confidence to quit. The role of self-efficacy in changing adverse behaviours, like smoking, has been highlighted in many studies and smokers with higher self-efficacy achieve a higher rate of tobacco cessation [44]. Shelley et al. (2010) showed that among Chinese-American smokers, the level of self-efficacy to quit smoking was strongly associated with cessation status. It was found that smokers who were strongly confident about quitting could quit successfully while those who were not strongly confident continued smoking [45].

In our study some of the participants mentioned a low level of self-efficacy to quit smoking however, they did express a strong intention to quit smoking. To help smokers to quit smoking, implementation intention maybe very effective because the unconscious cannot implement broad, vague intentions. Implementation intentions can translate goals and values into specific behavioural plans [46].

The role of other smokers, including family members and friends, in smoking initiation and maintenance or has been highlighted as a barrier of smoking cessation in this study. Research indicates that the social norms influence all stages of smoking behaviour [47]. Nguyent and others (2012) revealed that older smokers complied with smoking norms in their established social networks. When older smokers gave up smoking, this changed their relationships with other smokers in their social networks [48].

Withdrawal symptoms and nicotine craving were also found to be a significant barrier for older Greek-Australian smokers in attempts to quit. Anxiety, stress, loneliness, and putting on weight were the main withdrawal symptoms mentioned by our cohort of older Greek-Australian smokers, symptoms also noted in other populations of older smokers [10]. Smokers with a high degree of dependence can also present with low motivation due to their lack of confidence in their ability to succeed; they believe they are incapable of quitting and are afraid of suffering from the withdrawal syndrome which had undermined their previous attempts [49].

The interviews described here indicate that any attempt to assist older Greek-Australians to cease smoking will have to address both knowledge and attitudes. Misinformation and misunderstanding about the links between smoking and cancer causation must be directly addressed in this group and future research should identify credible educators whose ideas would resonate within the older Greek Australian community. Additionally, assistance with identification of the specific barriers to cessation experienced by each individual would require tailored support for quitting suggesting that the usual care population approaches (eg., Quitline use) may require targeted modification [50].

This study has a significant strength. To our knowledge, it is the first study conducted among older smokers in one of the biggest ethnic communities in Australia. This study had two limitations. The bilingual translator was a popular person and in some cases had previous contact with the participants. This issue might affect the quality of translations and speaking from her point of view. Another limitation of the study was related to the sampling. The participants were not selected randomly, so that selection bias may affect the result of the study.

The findings are important to health care professionals at all levels. It is important for both practitioners and policy makers to understand that smoking behaviour in older Greek-Australians is affected by many factors as mentioned in this paper. Additional studies could further investigate the effectiveness of smoking cessation intervention and counselling among older Greek smokers. It has been pointed out that every smoker’s behaviour can be investigated in the light of cultural belief and thus to change the behaviour a consideration of culture is an important factor. Smoking cessation strategies for this ethnic group should consider changing perceptions of what is socially acceptable behaviour.

## Conclusion

This study was conducted to understand the perspectives of older Greek–Australian smokers’ on smoking and the influences on their smoking behaviours. The analysis of the data revealed some new aspects of smoking behaviour among older people, in particular immigrants. It made clear that, although cultural beliefs and social values play an important role in smoking behaviour among older people, this behaviour is also influenced by individual factors such as knowledge and attitudes. When combined with cultural beliefs and events, the individual experiences of older smokers can make it doubly difficult to quit smoking. Health planners who are tailoring education programs to age groups need to consider the psychological barriers to the cessation of smoking. More research is needed on the barriers to quitting and on activities to educate smokers in this target group. The design of an intervention program that considers older smokers’ cultural perceptions in a field of individual influences, such as knowledge and attitudes, may shape the successful cessation of smoking among older immigrants in the future.

### Endnotes

^a^started with tobacco, but now smokes cigarettes. P6 quitted smoking due to getting cold.

^b^stopped smoking due to stay in hospital. P15 and 18 ceased smoking due to pregnancy.

## Abbreviations

CDC: Centers for disease control and prevention
NESB: Non-english speaking background
LGA: Local Government Area
GOCSA: Greek Orthodox Community Centre of South Australia
SPSS: Statistical package for the social sciences
SBREC: Social and Behavioural Research Ethic Committee
